# Supplementary material for: Modeling glioblastoma heterogeneity as a dynamic network of cell states
Source: Mol Syst Biol. 2021 Sep 16;17(9):e10105. doi: 10.15252/msb.202010105 (PMC8444284; doi:10.15252/msb.202010105)
Supplement: Supplementary file 5 — Source Data for Figure 3 [file MSB-17-e10105-s001.zip › Figure3A_sourcedata/GSEA_3065/nosigdb_state6.GseaPreranked.1622718443993/RICHARDS_NOWAKOWSKI_SCIENCE_2017_IPC_NEN1_UPREG_1.html]

Details for gene set RICHARDS\_NOWAKOWSKI\_SCIENCE\_2017\_IPC\_NEN1\_UPREG\_1[GSEA]

|  || Dataset | state6 |
| Phenotype | NoPhenotypeAvailable |
| Upregulated in class | na\_pos |
| GeneSet | RICHARDS\_NOWAKOWSKI\_SCIENCE\_2017\_IPC\_NEN1\_UPREG\_1 |
| Enrichment Score (ES) | 0.40379068 |
| Normalized Enrichment Score (NES) | 1.7938026 |
| Nominal p-value | 0.0 |
| FDR q-value | 0.0054517733 |
| FWER p-Value | 0.087 |
Table: GSEA Results Summary

  

Fig 1: Enrichment plot: RICHARDS\_NOWAKOWSKI\_SCIENCE\_2017\_IPC\_NEN1\_UPREG\_1      
 Profile of the Running ES Score & Positions of GeneSet Members on the Rank Ordered List

  

| PROBE | GENE SYMBOL | GENE\_TITLE | RANK IN GENE LIST | RANK METRIC SCORE | RUNNING ES | CORE ENRICHMENT || 1 | SOX4 |  |  | 3 | 0.375 | 0.0339 | Yes |
| 2 | TMSB10 |  |  | 5 | 0.330 | 0.0638 | Yes |
| 3 | RPS27 |  |  | 11 | 0.307 | 0.0913 | Yes |
| 4 | CCND2 |  |  | 32 | 0.269 | 0.1135 | Yes |
| 5 | RPL10 |  |  | 33 | 0.265 | 0.1377 | Yes |
| 6 | RPL34 |  |  | 41 | 0.251 | 0.1597 | Yes |
| 7 | HES6 |  |  | 44 | 0.248 | 0.1821 | Yes |
| 8 | SOX11 |  |  | 70 | 0.230 | 0.2002 | Yes |
| 9 | RPS14 |  |  | 72 | 0.229 | 0.2210 | Yes |
| 10 | EIF3H |  |  | 74 | 0.228 | 0.2416 | Yes |
| 11 | RPS21 |  |  | 77 | 0.226 | 0.2620 | Yes |
| 12 | H3F3A |  |  | 104 | 0.212 | 0.2784 | Yes |
| 13 | TMSB4X |  |  | 162 | 0.187 | 0.2889 | Yes |
| 14 | SRP14 |  |  | 181 | 0.182 | 0.3034 | Yes |
| 15 | GTF2I |  |  | 284 | 0.154 | 0.3057 | Yes |
| 16 | ELAVL3 |  |  | 321 | 0.148 | 0.3150 | Yes |
| 17 | CORO1C |  |  | 345 | 0.145 | 0.3256 | Yes |
| 18 | COMMD6 |  |  | 350 | 0.144 | 0.3382 | Yes |
| 19 | HNRNPH1 |  |  | 378 | 0.139 | 0.3478 | Yes |
| 20 | ATP6V1F |  |  | 407 | 0.135 | 0.3569 | Yes |
| 21 | SETD7 |  |  | 430 | 0.131 | 0.3663 | Yes |
| 22 | YWHAQ |  |  | 478 | 0.125 | 0.3723 | Yes |
| 23 | MEX3A |  |  | 660 | 0.109 | 0.3614 | Yes |
| 24 | FYTTD1 |  |  | 668 | 0.109 | 0.3705 | Yes |
| 25 | CD24 |  |  | 674 | 0.108 | 0.3798 | Yes |
| 26 | PHC2 |  |  | 727 | 0.103 | 0.3832 | Yes |
| 27 | NDUFA6 |  |  | 730 | 0.103 | 0.3924 | Yes |
| 28 | PDHA1 |  |  | 977 | 0.087 | 0.3720 | Yes |
| 29 | TLK2 |  |  | 1015 | 0.085 | 0.3754 | Yes |
| 30 | RPL7 |  |  | 1076 | 0.082 | 0.3760 | Yes |
| 31 | MARCKS |  |  | 1081 | 0.081 | 0.3829 | Yes |
| 32 | MIDN |  |  | 1145 | 0.078 | 0.3828 | Yes |
| 33 | KIF3C |  |  | 1150 | 0.078 | 0.3894 | Yes |
| 34 | ZNF462 |  |  | 1164 | 0.077 | 0.3950 | Yes |
| 35 | BAZ2B |  |  | 1208 | 0.075 | 0.3969 | Yes |
| 36 | RND3 |  |  | 1213 | 0.075 | 0.4032 | Yes |
| 37 | CHTOP |  |  | 1267 | 0.073 | 0.4038 | Yes |
| 38 | POU3F3 |  |  | 1370 | 0.068 | 0.3983 | No |
| 39 | TMEM158 |  |  | 1598 | 0.061 | 0.3776 | No |
| 40 | PREX1 |  |  | 1790 | 0.054 | 0.3605 | No |
| 41 | SORBS1 |  |  | 1888 | 0.051 | 0.3539 | No |
| 42 | ENC1 |  |  | 1970 | 0.049 | 0.3490 | No |
| 43 | PSMA7 |  |  | 2029 | 0.047 | 0.3466 | No |
| 44 | FAM13B |  |  | 2062 | 0.046 | 0.3471 | No |
| 45 | EZR |  |  | 2077 | 0.046 | 0.3496 | No |
| 46 | FOXJ3 |  |  | 2106 | 0.045 | 0.3505 | No |
| 47 | HBP1 |  |  | 2202 | 0.042 | 0.3433 | No |
| 48 | MLLT11 |  |  | 2335 | 0.038 | 0.3315 | No |
| 49 | DCAF13 |  |  | 2384 | 0.036 | 0.3293 | No |
| 50 | CSTB |  |  | 2642 | 0.030 | 0.3024 | No |
| 51 | ST8SIA1 |  |  | 2714 | 0.029 | 0.2968 | No |
| 52 | NFIB |  |  | 2762 | 0.028 | 0.2939 | No |
| 53 | FMR1 |  |  | 2814 | 0.026 | 0.2904 | No |
| 54 | CORO2B |  |  | 2932 | 0.023 | 0.2790 | No |
| 55 | RAB12 |  |  | 2952 | 0.023 | 0.2789 | No |
| 56 | PLXNA2 |  |  | 3045 | 0.021 | 0.2702 | No |
| 57 | AGTPBP1 |  |  | 3046 | 0.021 | 0.2721 | No |
| 58 | PRKX |  |  | 3081 | 0.020 | 0.2701 | No |
| 59 | PPP4R1 |  |  | 3085 | 0.020 | 0.2716 | No |
| 60 | NRN1 |  |  | 3099 | 0.020 | 0.2719 | No |
| 61 | SRSF6 |  |  | 3114 | 0.020 | 0.2721 | No |
| 62 | NUMBL |  |  | 3153 | 0.019 | 0.2694 | No |
| 63 | SMARCD3 |  |  | 3281 | 0.016 | 0.2562 | No |
| 64 | SPIRE1 |  |  | 3332 | 0.015 | 0.2517 | No |
| 65 | TXNRD1 |  |  | 3380 | 0.014 | 0.2476 | No |
| 66 | CDC5L |  |  | 3466 | 0.012 | 0.2388 | No |
| 67 | SCHIP1 |  |  | 3493 | 0.011 | 0.2368 | No |
| 68 | KPNA4 |  |  | 3506 | 0.011 | 0.2365 | No |
| 69 | MYNN |  |  | 3699 | 0.007 | 0.2149 | No |
| 70 | NREP |  |  | 3707 | 0.007 | 0.2147 | No |
| 71 | RAD23B |  |  | 3871 | 0.003 | 0.1962 | No |
| 72 | LZTS1 |  |  | 3897 | 0.002 | 0.1936 | No |
| 73 | FDFT1 |  |  | 3979 | 0.001 | 0.1843 | No |
| 74 | ARL17A |  |  | 3989 | 0.001 | 0.1833 | No |
| 75 | RYBP |  |  | 4056 | -0.000 | 0.1757 | No |
| 76 | RAD1 |  |  | 4196 | -0.003 | 0.1600 | No |
| 77 | CASK |  |  | 4430 | -0.007 | 0.1338 | No |
| 78 | THSD7A |  |  | 4486 | -0.008 | 0.1282 | No |
| 79 | USP48 |  |  | 4651 | -0.012 | 0.1103 | No |
| 80 | MARK3 |  |  | 4659 | -0.012 | 0.1106 | No |
| 81 | MEIS2 |  |  | 4714 | -0.013 | 0.1055 | No |
| 82 | IDS |  |  | 4847 | -0.015 | 0.0917 | No |
| 83 | TRIM37 |  |  | 4852 | -0.015 | 0.0927 | No |
| 84 | GNG4 |  |  | 4886 | -0.016 | 0.0903 | No |
| 85 | ASF1A |  |  | 4888 | -0.016 | 0.0917 | No |
| 86 | CEP78 |  |  | 5239 | -0.023 | 0.0534 | No |
| 87 | PRDX1 |  |  | 5508 | -0.029 | 0.0251 | No |
| 88 | STAG1 |  |  | 5538 | -0.029 | 0.0244 | No |
| 89 | TAGLN3 |  |  | 5582 | -0.030 | 0.0222 | No |
| 90 | THBS1 |  |  | 5670 | -0.032 | 0.0151 | No |
| 91 | PTPRK |  |  | 5681 | -0.032 | 0.0169 | No |
| 92 | GLCE |  |  | 5702 | -0.032 | 0.0175 | No |
| 93 | PSME1 |  |  | 5806 | -0.034 | 0.0088 | No |
| 94 | DHX9 |  |  | 5900 | -0.036 | 0.0013 | No |
| 95 | POU3F2 |  |  | 6000 | -0.038 | -0.0066 | No |
| 96 | CYCS |  |  | 6090 | -0.040 | -0.0132 | No |
| 97 | MEF2A |  |  | 6220 | -0.043 | -0.0242 | No |
| 98 | PTPRS |  |  | 6439 | -0.048 | -0.0450 | No |
| 99 | AUTS2 |  |  | 6594 | -0.053 | -0.0579 | No |
| 100 | TUBB |  |  | 6820 | -0.059 | -0.0785 | No |
| 101 | ITPR2 |  |  | 7079 | -0.067 | -0.1021 | No |
| 102 | QSER1 |  |  | 7135 | -0.069 | -0.1022 | No |
| 103 | ELOVL5 |  |  | 7151 | -0.069 | -0.0976 | No |
| 104 | LRCH3 |  |  | 7185 | -0.071 | -0.0950 | No |
| 105 | CLIP1 |  |  | 7187 | -0.071 | -0.0887 | No |
| 106 | MLLT3 |  |  | 7230 | -0.072 | -0.0870 | No |
| 107 | RICTOR |  |  | 7252 | -0.073 | -0.0827 | No |
| 108 | ZNF704 |  |  | 7300 | -0.074 | -0.0814 | No |
| 109 | GRIA2 |  |  | 7312 | -0.075 | -0.0758 | No |
| 110 | MECP2 |  |  | 7429 | -0.079 | -0.0820 | No |
| 111 | ZDHHC17 |  |  | 7468 | -0.080 | -0.0791 | No |
| 112 | CHMP5 |  |  | 7588 | -0.085 | -0.0851 | No |
| 113 | SYNE2 |  |  | 7598 | -0.086 | -0.0783 | No |
| 114 | PGAP1 |  |  | 7701 | -0.091 | -0.0817 | No |
| 115 | LRP8 |  |  | 7752 | -0.093 | -0.0790 | No |
| 116 | CTR9 |  |  | 7759 | -0.093 | -0.0712 | No |
| 117 | TCF25 |  |  | 7795 | -0.095 | -0.0665 | No |
| 118 | GLS |  |  | 7856 | -0.099 | -0.0644 | No |
| 119 | GPM6A |  |  | 7875 | -0.100 | -0.0574 | No |
| 120 | SORBS2 |  |  | 7917 | -0.102 | -0.0528 | No |
| 121 | ANK3 |  |  | 7938 | -0.103 | -0.0457 | No |
| 122 | ZNF292 |  |  | 8071 | -0.111 | -0.0508 | No |
| 123 | XPO1 |  |  | 8216 | -0.123 | -0.0562 | No |
| 124 | PDE1C |  |  | 8263 | -0.128 | -0.0499 | No |
| 125 | SLC4A7 |  |  | 8273 | -0.129 | -0.0392 | No |
| 126 | TUBA1C |  |  | 8365 | -0.142 | -0.0367 | No |
| 127 | UGGT2 |  |  | 8400 | -0.146 | -0.0273 | No |
| 128 | IGF2BP2 |  |  | 8487 | -0.158 | -0.0228 | No |
| 129 | CWC27 |  |  | 8577 | -0.180 | -0.0166 | No |
| 130 | NTM |  |  | 8612 | -0.196 | -0.0027 | No |
| 131 | TMEM59 |  |  | 8723 | -0.261 | 0.0084 | No |
Table: GSEA details [plain text format]

  

Fig 2: RICHARDS\_NOWAKOWSKI\_SCIENCE\_2017\_IPC\_NEN1\_UPREG\_1: Random ES distribution      
 Gene set null distribution of ES for **RICHARDS\_NOWAKOWSKI\_SCIENCE\_2017\_IPC\_NEN1\_UPREG\_1**
